# Electrochemical Degradation of Reactive Black 5 using two-different reactor configuration

**DOI:** 10.1038/s41598-020-61501-5

**Published:** 2020-03-11

**Authors:** Tamara Droguett, Julia Mora-Gómez, Montserrat García-Gabaldón, Emma Ortega, Sergio Mestre, Gerardo Cifuentes, Valentín Pérez-Herranz

**Affiliations:** 1 IEC Group, ISIRYM, Universitat Politècnica de València, Camí de Vera s/n, 46022, València, P.O. Box 22012, E-46071 Spain; 2 Instituto Universitario de Tecnología Cerámica, Universitat Jaume I, Castellón, Spain; 3 Depto. de Ingeniería Metalúrgica, Facultad de Ingeniería, Universidad de Santiago de Chile (USACH), Santiago de Chile, Chile

**Keywords:** Pollution remediation, Environmental impact

## Abstract

Novel Sb-doped SnO_2_ ceramic electrodes sintered at different temperatures, are applied to the degradation of Reactive Black 5 in both divided and undivided electrochemical reactors. In the undivided reactor the discoloration of the solution took place via the oxidation of RB5 dye, without the corresponding reduction in the chemical oxygen demand for the ceramic electrodes. However, in the divided one, it was possible to achieve the discoloration of the solution while at the same time decreasing the chemical oxygen demand through the ·OH-mediated oxidation, although the chemical oxygen demand degradation took place at a slower rate.

## Introduction

Azo dyes are widely employed in textile manufacture, which is one of the most polluting sectors within the different human activities. A considerable amount of the dye (between 1–15%) is discharged into wastewaters after the dyeing process^1^. Reactive Black 5 (RB5), is considered the best option for cotton and other cellulose fibers^2^, and consequently, is the azo dye with the largest consumption rate^2^.

The release of colored wastes is not only an aesthetical aspect, but also avoids the penetration of light, which affects the biological processes. Moreover, many dyes are susceptible of generating toxicity in the aquatic organisms as well as in humans^3^. In addition, the aromatic structures of these chemicals are very refractory and, therefore, resistant to be degraded in wastewater treatment plants since they remain unaffected towards sunlight, oxidizing agents and microorganisms^4^.

The elimination and degradation of RB5 has been reported in the scientific literature using membrane separation techniques^5–7^, electrocoagulation^2,8^, photocatalysis^9–11^, biodegradation^12,13^, advanced oxidation processes^14,15^, electrochemical reduction and oxidation^16–18^ or combined methods^19–21^. Membrane processes, electrocoagulation, direct precipitation or adsorption, just change the contamination from one phase to another. Chemical oxidation using agents such as chlorine produces organochlorine compounds considered very toxic. Photooxidation needs the addition of chemicals, and causes a secondary pollution. The biological degradation is less effective than other methods because of the inhibition of the bacterial activity due to the toxicity of dye^22^.

Electrochemical methods are able of mineralizing the dyes without the addition of chemicals. Besides this advantage, they can be coupled to sources of renewable energy with the aim of decreasing the operational costs^23,24^. The electrochemical oxidation mainly operates in two different pathways. In the direct oxidation, the contaminant is directly oxidized on the anodic surface; whilst in the indirect oxidation, the transfer of electrons is mediated by oxidant species^25^ such as the hydroxil radicals. The named “active” anodes present low oxygen evolution overpotential (Pt, IrO_2_ and RuO_2_) and possess chemisorbed ·OH radicals, only permitting the partial oxidation of the organic matter. On the contrary, “nonactive” anodes with high oxygen evolution overpotential produce physisorbed ·OH radicals favoring complete mineralization^26^. One of the most widely used anode for the electrochemical degradation of pollutants is the BDD, which has the highest overpotential for oxygen evolution^27–29^. However, these anodes are expensive and their manufacture is complicated, due to the difficulty of finding cheaper materials for the deposition of the diamond layer^30^.

Sb-doped SnO_2_ anodes, which possess a medium to high oxidation power with an oxygen evolution potential of about 1.9 V vs. SHE^26^, have resulted to be highly effective for the electrooxidation of organics^31–37^. Another advantages associated to these electrodes are their fabrication easiness and low cost. However, their major drawback is their low stability when employed as anodes^38^. This is why its use as a massive electrode is not common and SnO_2_ is normally used as a coating on a metal substrate.

On the hand, ceramic electrodes are being of great importance, as an example is their use in fuel cells working at high temperature, molten salt processes^39,40^, and in electrochemical wastewater treatment^41–44^. Ceramic materials are in their most stable, completely oxidized form, and therefore, unlikely to be further oxidized. Moreover, because of their porous structure, they present a large specific surface area, providing active sites for pollutants adsorption and reaction^45^. Microporous ceramic electrodes could also be used as a reactive membrane to filter the foulants, while acting as an effective electrode to destroy organic foulants, integrating physical separation with electrochemical oxidation.

In a previous study^46^, new microporous Sb-doped SnO_2_ ceramic electrodes at different sintering temperatures were tested to be used as anodes in electrochemical degradation processes. It was found that the sintered electrode at the lowest temperature (1050 °C) presented a greater potential for oxygen discharge, this value being higher than the Pt electrode (active anode) but lower than the BDD (nonactive anode). In addition, electro-oxidation tests of Norfloxacin (NOR), a widely used antibiotic, were carried out with these electrodes in galvanostatic and potentiostatic mode. The results obtained in terms of degradation and mineralization were satisfactory. In another study^47^, the influence of the reactor configuration (divided and undivided) was studied, and the authors showed that in the divided reactor the degradation of the organic compound was greater than in the undivided one, mainly due to the fact that the membrane prevented the intermediate products formed during the NOR oxidation process from being reduced on the cathode.

The aim of the present work is to corroborate the results obtained previously with these microporous Sb-doped SnO_2_ ceramic electrodes, verifying their versatility for treating different organic compounds, specifically the RB5 dye, in an undivided and a divided electrochemical cell. In addition, to ensure the electrochemical stability of these electrodes, accelerated life tests under high current density conditions were conducted.

## Methods

### Electrodes characterization and service life tests

The manufacture of the Sb-doped SnO_2_ ceramic electrodes sintered at different temperatures (1050 °C, 1150 °C and 1250 °C) is fully described in detail previously^46,47^. With respect to the accelerated life tests, they consist in the application of a constant current density (100 mA·cm^−2^) during 24 h in a 0.5 M H_2_SO_4_ solution. The working electrode was the ceramic electrode under study, in the presence of Pt as counter electrode, and Ag/AgCl as reference electrode.

The potential of the working electrode is periodically monitored, and the electrode is considered deactivated when its potential increases 5 V from its initial value^48,49^. If the potential of the working electrode did not change after this period of time, the applied current density was increased by 100 mA·cm^−2^ and the cycle was repeated several times.

### Electrolysis experiments

The experimental arrangement of both the divided and undivided reactors was previously described in detail^47^. The anode was either a ceramic electrode or a Boron-doped diamond (BDD) electrode (from NeoCoat SA, Switzerland), which is a well-studied anode. Electrolyses experiments were carried out galvanostatically with an Autolab PGSTAT302N potentiostat/galvanostat at applied current values between 5 and 15 mA·cm^−2^.

**Figure 1:**
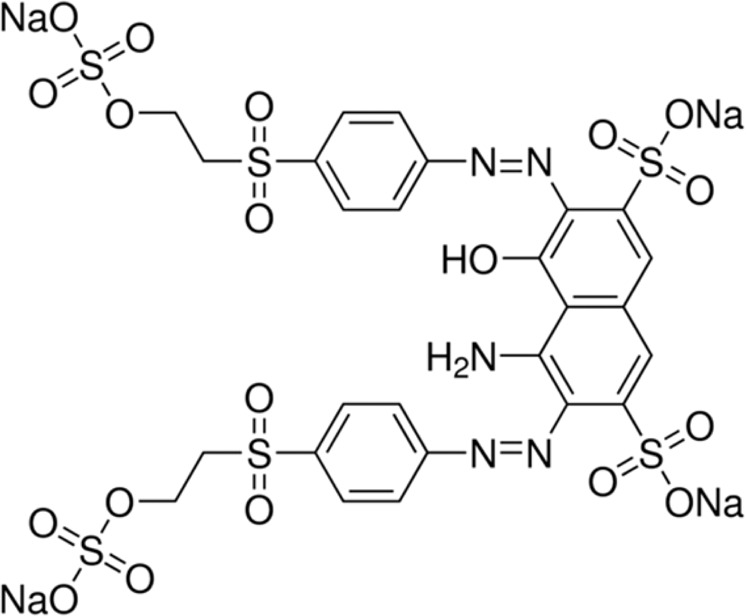
Structure of Reactive Black 5 (C.I. No. 20505; M.W. = 991.8).

RB5, whose structure is given in Fig. 1, was purchased from Sigma–Aldrich and Na_2_SO_4_ was used as supporting electrolyte (from Panreac). Solutions of 100 mg·L^−1^ of RB5 and 0.1 mol·L^−1^ of Na_2_SO_4_ were tested in both type of reactors. All experiments were performed under stirring and at room temperature during 5 hours.

### Analytical measurements

Every 30 min samples were extracted from the electrochemical reactor, and different operation parameters such as current density or cell voltage were monitored. The discoloration rate and the chemical oxygen demand were measured from every extracted sample. UV-visible spectra were analyzed between 200 and 800 nm using a Unicam UV4-200 UV/VIS Spectrometer. The mineralization of the dye was measured from the diminution of the chemical oxygen demand (COD) of every sample as a function of time, determined using the dichromate method.

The discoloration of the RB5 solutions was measured from the relative absorbance decrease at 595 nm, which is the maximum visible wavelength (λ_max_), according to the following expression^50^:

1 $$Relative\,absorbance=\frac{Ab{s}_{t}}{Ab{s}_{0}}$$

where Abs_t_ is the absorbance for a given time t, and Abs_0_ is the initial absorbance value.

The current efficiency (ϕ) of the RB5 oxidation process was estimated from the expression^26^:

2 $$\phi =FV\left(\frac{CO{D}_{0}-CO{D}_{t}}{8{\int }_{0}^{t}I(t)dt}\right)$$

where COD_0_ is the initial chemical oxygen demand and COD_t_ is the same parameter for a given time t, I the current applied, F the constant of Faraday and V is the volume of the reactor.

## Results and Discussion

### Service life tests

**Figure 2:**
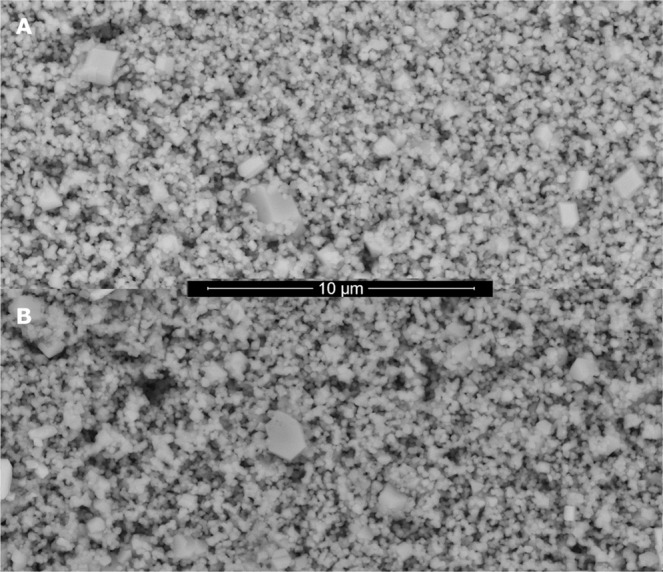
SEM images of the as-prepared ceramic electrode sintered at 1050 °C (**A**) and after the service life tests (**B**).

**Figure 3:**
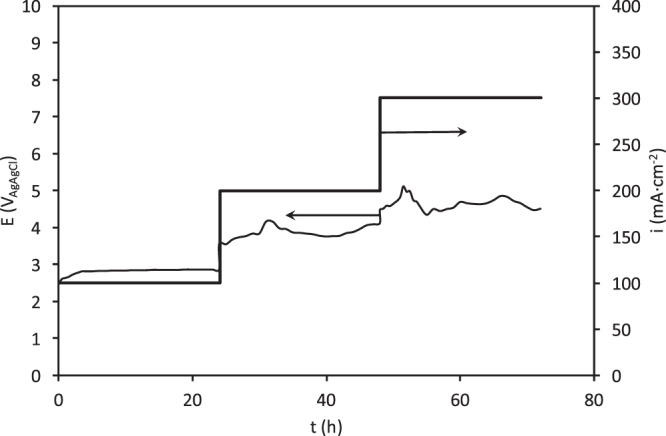
Accelerated service life test of the ceramic electrode sintered at 1050 °C in 0.5 M H_2_SO_4_.

In Fig. 2, SEM images of the as-prepared ceramic electrode sintered at 1050 °C (A) and after the service life tests (B) are shown, while Fig. 3 illustrates the accelerated service life test of the ceramic electrode sintered at 1050 °C in 0.5 M H_2_SO_4_. At the imposed applied current densities, the oxygen evolution was the only reaction that takes place, and the gas evolution was observed. There were no visible changes in the electrode throughout the experiment as was confirmed by SEM images. In fact, the electrode retains the structure of the freshly prepared one as can be seen in Fig. 2(B). Overall, it is clear that the electrolysis performed in the conditions of Fig. 3 did not cause any significant damage on the electrode. In fact, the electrode was subsequently reused for another electrolysis experiments.

Figure 3 shows the evolution of the electrode potential during this service life test. When the electrolysis was commenced at an applied current density (i) of 100 mA·cm^−2^, the initial electrode potential was about 2.5 V but within the three first hours it slowly increased to 2.85 V and thereafter remained almost constant. After 24 hours of electrolysis, i rose up to 200 mA·cm^−2^. At this applied current density, the electrode potential took an initial value of 3.5 V, and then it increased slowly with time until reaching an average value of about 3.8 volts. Under these conditions the electrode potential oscillated around this average value, and reached a maximum value of 4.1 V in the sixth hour. When i increased up to 300 mA·cm^−2^, the electrode potential oscillated around 4.5 V during the 24 hours of electrolysis. In this case, the potential oscillations were greater than for 200 mA·cm^−2^, and several potential peaks of approximately 5 V were observed. These potential peaks observed for i values of 200 and 300 mA·cm^−2^ are associated with the formation of oxygen bubbles that block the electrode surface and cause an increase in the electrode potential. When the bubbles leave the electrode surface, the potential returns to its initial value. This phenomenon occurs more often at higher applied current densities. Similar results were obtained for the electrodes sintered at the other temperatures (not shown).

Different results regarding with the service life of SnO_2_-Sb based anodes are found in the literature. A service life value of about 12 h for Ti/SnO_2_-Sb_2_O_5_ anodes was obtained at 100 mA·cm^−2^ in 1 M H_2_SO_4_^51^, while other works reported no change over 48 days for the same type of anodes^38^. On the other hand, service lifetime greater than 30 days for Ti/SnO_2_-Sb_2_O_5_ was obtained by other authors^52^, whilst lower values of service lifetime from 6.4 h to 42 h for the same anodes were also reported^53^. Another study found only 10 min for Ti/SnO_2_-Sb_2_O_5_ anodes^54^, and Chen and Nigro^55^ reported values from 0.68 to 60 hours depending on the substrate. These large variations in the service lifetime reported in the literature may be related to the method of preparing the electrodes.

Hence, taking into consideration the results obtained by the lifetime service tests and considering that no change was observed in the microstructure of the different electrodes tested, it is inferred that the Sb-SnO_2_ ceramic electrodes under study are a good alternative to conventional SnO_2_ electrodes.

### Electrolysis experiments

#### Undivided reactor

**Figure 4:**
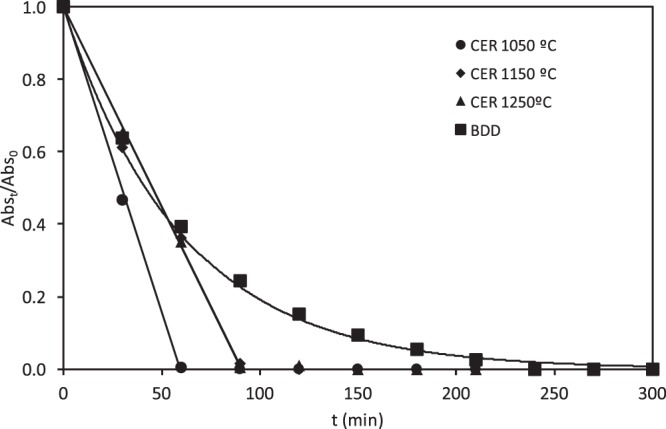
Evolution of the relative absorbance (at 595 nm) with the electrolysis time for the electrochemical degradation of RB5 with the ceramic electrodes sintered at different temperatures and the BDD electrode in the undivided reactor.

Figure 4 presents the relative absorbance measured at 595 nm (where a maximum absorbance is obtained) over time in the undivided reactor, for the ceramic electrodes sintered at different temperatures and at 15 mA·cm^−2^. The results are compared with those obtained with the BDD electrode at the same i value. The electrochemical degradation of RB5 using either the ceramic or the BDD electrodes led to discoloration of the solution as the electrolysis progressed. However, the discoloration kinetics was different depending on the electrode. With the ceramic electrodes, a rapid discoloration of the solution was achieved in less than 90 minutes and in this period, the relative absorbance decreased linearly with time until complete discoloration. In contrast, the discoloration of the solution with the BDD electrode was slower, and the relative absorbance decreased exponentially with time, showing a first order discoloration kinetics.

Different reasons could justify the differences in discoloration observed between both types of electrodes. On one hand, the surface structure of the ceramic electrodes is different from that of the BDD one. Thus, for the same geometric surface area, the ceramic electrodes present a larger specific surface area due to their microporous structure. This causes that for the same applied current density, the electrode potential reached with the ceramic electrodes is less anodic than that reached with the BDD electrode. In fact, for the same value of i (15 mA·cm^−2^), the average electrode potential values obtained with each electrode were 2.4 V for the ceramic electrode sintered at 1050 °C, 2.6 V for those sintered at 1150 and 1250 °C and 3 V for the BDD electrode. Therefore, higher discoloration efficiency should be achieved with the ceramic electrode sintered at 1050 °C.

Comparing these results with those obtained previously for the removal of NOR with the same electrodes^46^, it is observed that using the ceramic electrode of the lowest sintering temperature (1050 °C) the removal of both compounds (NOR and RB5) is greater. This fact is because this electrode presents the highest potential for the oxygen discharge.

**Figure 5:**
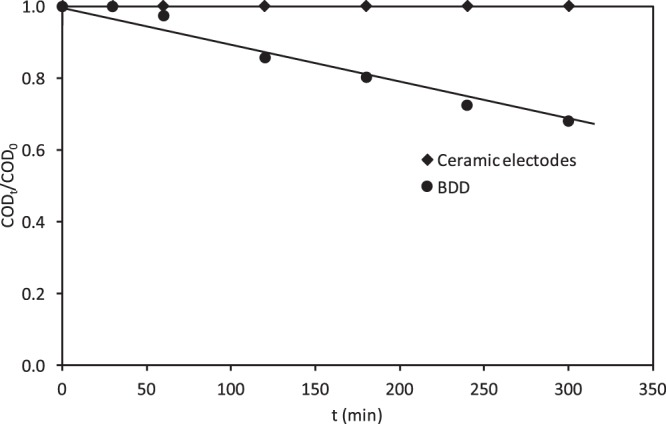
Evolution of the relative COD removal with the electrolysis time for the ceramic electrodes sintered at different temperatures and the BDD electrode in the undivided reactor.

Figure 5 shows the COD versus the electrolysis time for the ceramic and the BDD electrodes. A maximum COD decrease of 35% is achieved after 5 hours of electrolysis with the BDD electrode, whereas for the same electrolysis time, there was no decrease of the COD with the ceramic ones. This low efficiency in COD removal may be due to the formation of different intermediates of the RB5 dye, which are not subsequently mineralized. Some of these intermediates are quite stable and may be more toxic than the original dye^56,57^. During the degradation of the RB5, the aromatic ring structure can be one of the intermediates.

**Figure 6:**
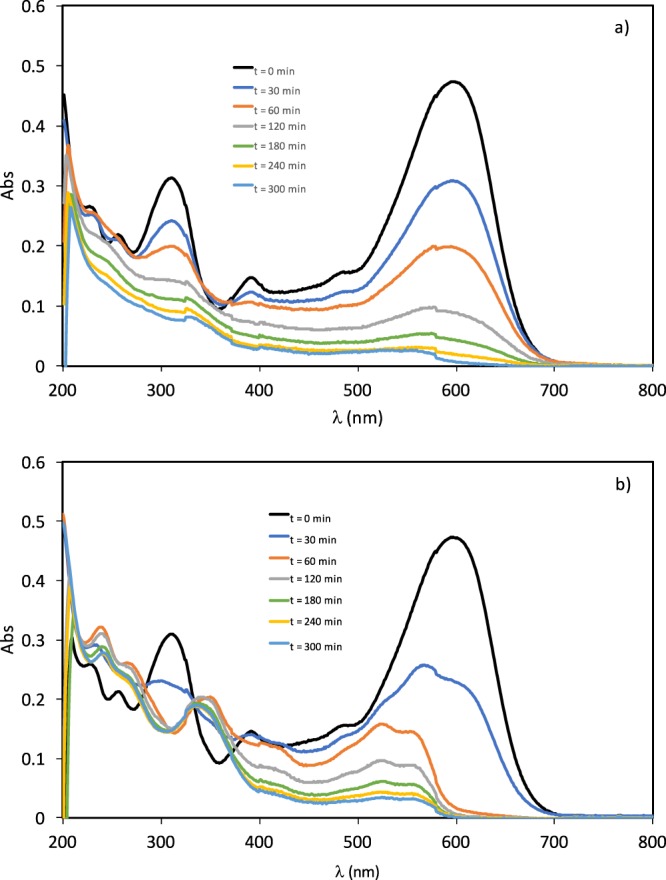
Variation of the UV-visible spectra of a 100 mg·L^−1^ RB5 solution with the electrolysis time in an undivided reactor. a) BDD electrode. b) ceramic electrode sintered at 1050 °C.

UV-visible absorption spectra of the 100 mg·L^−1^ RB5 solution at different electrolysis times are shown in Fig. 6a) for the BDD electrode and in Fig. 6b) for the ceramic electrode sintered at 1050 °C. Before the electrolysis, the spectra of RB5 shows five bands: one in the visible region (at 595 nm) and others in the ultraviolet region (229, 254, 310 and 391 nm). The peak at 595 nm is related to the chromophoric azo group (-N=N-), while the peaks at 254 and 310 nm are associated with benzene and naphthalene rings^11,19,58^. As can be seen in Fig. 6a), all the peaks decrease as the electrolysis proceeded. The diminution of the peak at 595 nm suggests that the chromophore groups are broken down easily. On the other hand, the decrease in absorbance in the UV region is lower, and consequently, the aromatic structures remain more stable. This fact explains the low COD removal rate shown in Fig. 5.

For the ceramic electrode (Fig. 6b)), it was observed a progressive decrease of the absorption band at 595 nm, along with a shift towards lower wavelengths (at 523 nm). This was evidenced by a color change in the solution from the initial characteristic blue color of the RB5 solution to a final reddish color. Jager *et al*.^59^ obtained an identical UV-Visible spectrum for RB5, as well as the same reddish color, that was attributed to the presence of an isoxazole derivative formed by the cyclization of the original molecule in the oxidation process. After 5 h of treatment, the absorbance in the UV region remained almost constant with time which suggests the persistence of aromatic rings.

**Figure 7:**
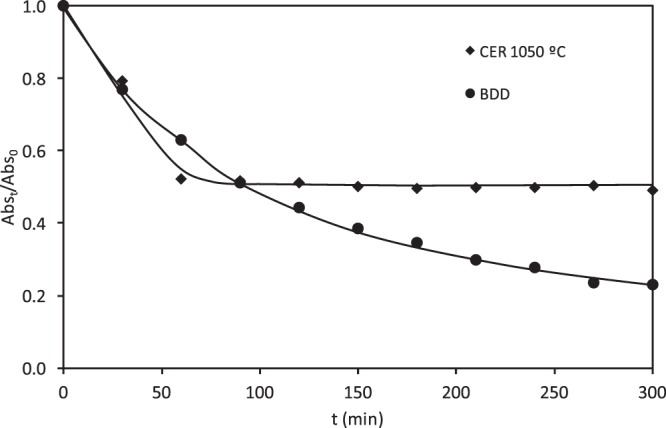
Evolution of the relative absorbance (at 310 nm) with the electrolysis time for the electrochemical degradation of RB5 with the ceramic electrode sintered at 1050 ªC and the BDD electrode in the undivided reactor.

In order to compare the discoloration rate (595 nm) shown in Fig. 4 and the aromatic fragment degradation (310 nm), the evolution with time of the relative absorbance measured at 310 nm for the ceramic electrode sintered at 1050 °C and the BDD electrode is shown in Fig. 7 at 15 mA·cm^−2^. Comparing Figs. 4 and 7, it is inferred that discoloration of RB5 is faster, while the degradation of benzenic or naphtalenic cycles is much more difficult. For the ceramic electrode, the band at 310 nm decreases rapidly reaching a plateau at 60 min and then remains constant with time, while for the BDD electrode this band decreases continuously with time, but at a lower rate than the band at 595 nm. The observed differences between the ceramic electrodes and the BDD electrode in the absorption band at 310 nm would explain why the ceramic electrodes do not practically present COD removal, and why the discoloration of the BDD electrode is faster than the degradation of the benzenic or naphtalenic rings.

Therefore, in this type of reactor, the BDD anode is more efficient than the ceramic one as the BDD electrode reduced a 35% of the COD, while the ceramic anodes were not active for COD removal. Using the BDD electrode the N=N bonds of the azo groups are attacked first, causing discoloration unlike what happens in the ceramic electrode, in which the RB5 molecule undergoes an intramolecular cyclization causing changes in the bands of the UV-VIS spectra. The aromatic ring structures are more stable, and consequently, the COD removal is slower. On the other hand, it can be concluded that by-products formed with the ceramic electrode are more difficult to degrade and therefore a decrease in COD values is not observed.

#### Divided reactor

According to the previous results, it seems necessary to perform the electrochemical oxidation of the RB5 in a divided reactor. In this case, the RB5 solution was placed in the anodic chamber, and the use of a membrane between the compartments of the reactor is justified in order to prevent the reduction of the electrogenerated oxidizing species and the oxidized organic compounds. Electrolysis experiments were performed with the ceramic electrode sintered at 1050 °C under three values of i (5, 10 and 15 mA·cm^−2^).

**Figure 8:**
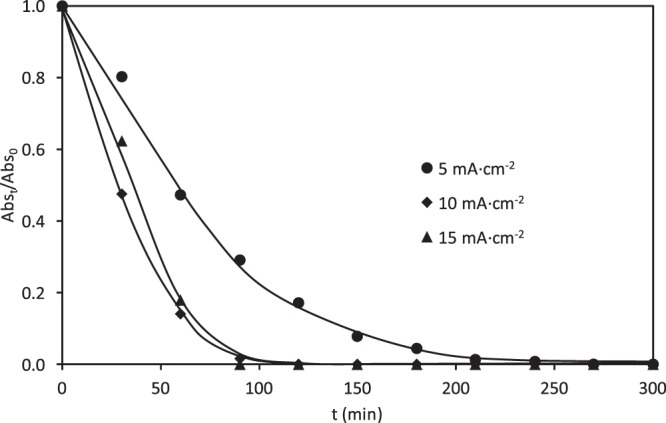
Effect of the applied current density on the evolution of the relative absorbance (at 595 nm) with the electrolysis time for the electrochemical degradation of RB5 with the ceramic electrode sintered at 1050 °C in the divided reactor.

**Figure 9:**
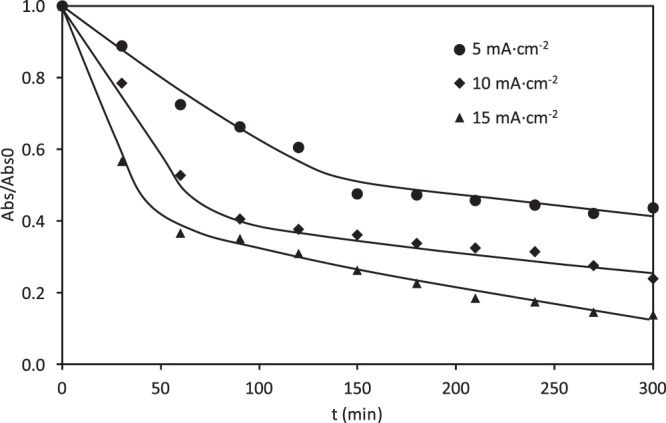
Effect of the applied current density on the evolution of the relative absorbance (at 310 nm) with the electrolysis time for the electrochemical degradation of RB5 with the ceramic electrode sintered at 1050 °C in the divided reactor.

The variation of the relative absorbance with time of a 100 mg·L^−1^ RB5 solution at 595 and 310 nm is presented in Figs. 8 and 9, respectively. Comparing both figures, it can be concluded that higher removal efficiency for RB5 was achieved at all applied current densities in the visible region. The discoloration at 595 nm after 90 min of electrolysis was 71%, 98% and 100% for 5, 10 and 15 mA·cm^−2^ respectively. The disappearance of the absorption band of 595 nm beyond 90 min at the highest current density may be attributed to the action of the ·OH radicals.

**Figure 10:**
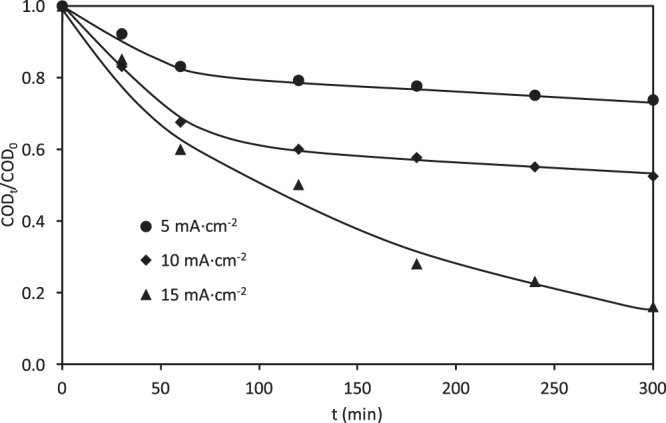
Effect of the applied current density on the evolution of the relative COD removal with the electrolysis time for the electrochemical degradation of RB5 with the ceramic electrode sintered at 1050 °C in the divided reactor.

However, as can be seen in Fig. 9, lower degradation efficiencies were obtained in the absorption band of 310 nm, which indicates that the aromatic structures of the RB5 molecule are more stable towards electrochemical degradation. However, unlike what happened in the undivided reactor with the same ceramic electrodes, where the absorption band of 310 nm remained constant after 60 minutes of electrolysis (Fig. 7), in this case, the relative absorbance at 310 nm diminishes rapidly until 60 minutes of electrolysis, and then it continues decreasing more slowly. Comparing Figs. 8 and 9, it is inferred that the relative absorbance decreases more slowly at 310 nm, which is reflected by a low mineralization degree of the dye. This fact can be verified in Fig. 10, which shows the evolution of the relative COD over time for the three applied current densities under study. In these experiments, a 30, 50 and 80% of the COD removal was recorded at the end of the electrolysis at 5, 10 and 15 mA·cm^−2^, respectively. The change in the COD removal follows a similar trend to that observed for the relative absorbance at 310 nm (Fig. 9). Similar differences between color and COD removal have been obtained by other authors when studying the degradation of dyes by electrocoagulation^20,21^, electrochemical oxidation^30,60–65^, photocatalysis^9,11^ photo-Fenton^19,57^ or biological methods^13,66^.

The oxidation of organic compounds on SnO_2_–Sb_2_O_5_ electrodes proceeds according to the following reaction^67–69^:

3 $$Sn{O}_{2}+{H}_{2}O\to Sn{O}_{2}(\,\cdot \,OH)+{H}^{+}+{e}^{-}$$

SnO_2_(·OH) can react with the organic compounds (R) to produce CO_2_, H_2_O, etc. though reaction (4) or be further oxidized to generate O_2_ gas through reaction (5):

4 $$Sn{O}_{2}(\cdot OH)+R\to Sn{O}_{2}+m\,C{O}_{2}+n\,{H}_{2}O+{H}^{+}+{e}^{-}$$

5 $$Sn{O}_{2}(\cdot OH)\to Sn{O}_{2}+\frac{1}{2}{O}_{2}+{H}^{+}+{e}^{-}$$

**Figure 11:**
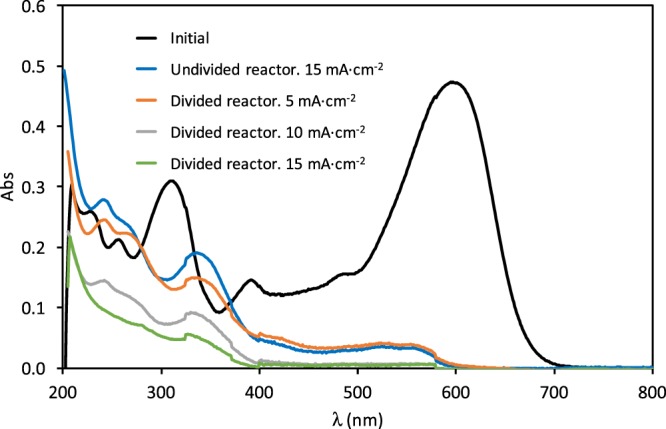
UV-visible absorption spectra of a 100 mg·L^−1^ RB5 solution during degradation with the ceramic electrode sintered at 1050 °C. Comparison of the divided and undivided reactors.

UV-visible absorption spectra of a 100 mg·L^−1^ RB5 solution before and after the treatment at the three applied current densities under study are presented in Fig. 11, and are compared with the spectra obtained in the undivided reactor at 15 mA·cm^−2^. Before the treatment, UV-visible spectra of RB5 show the characteristic absorption bands commented previously. When the solution was treated in the divided reactor, the absorption peak present at 595 nm disappears. Besides, the UV band at 310 nm decreased, although at a slower rate. During the treatment of the RB5 solution in the divided reactor, the absorbance values diminished all over the spectral window, unlike what happens in the undivided reactor.

**Figure 12:**
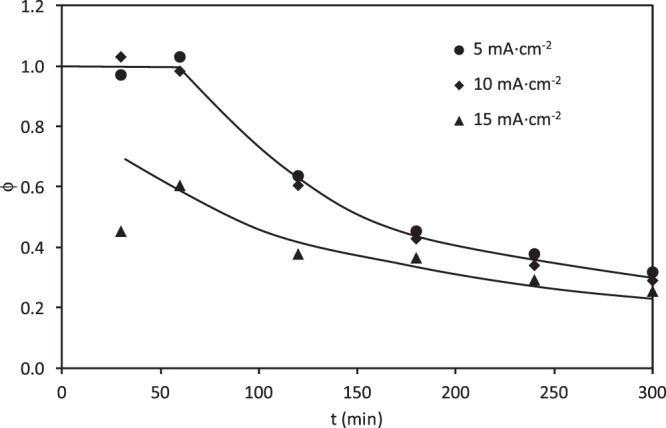
Effect of the applied current density on the evolution of CE with the electrolysis time for the electrochemical degradation of RB5 with the ceramic electrode sintered at 1050 °C in the divided reactor.

The evolution of the current efficiency (ϕ) with time, calculated from Eq. (2), is presented in Fig. 12 for the electrolysis of a 100 mg·L^−1^ RB5 solution. At 5 and 10 mA·cm^−2^, ϕ values of 100% were obtained during the first hour of electrolysis, although low efficiencies in the degradation of the COD were obtained in this period of time (Fig. 10). For these i values, after the first hour of electrolysis, ϕ decreased exponentially with time until reaching values close to 30%. However, at 15 mA·cm^−2^, ϕ decreased exponentially with time from the beginning of the electrolysis experiments, and the values of ϕ achieved were lower than those obtained at 5 and 10 mA·cm^−2^ for every time, even though a greater efficiency in COD removal was obtained at this higher applied current density.

According to the literature^26^, hydroxyl radicals are the intermediates for reactions (4) and (5), oxidation of organic matter and the side oxygen evolution reaction, respectively. If the mineralization process is controlled by the transport of the pollutant from the solution to the anode, two situations can be distinguished. When i is lower than the limiting current value corresponding to the initial concentration of organic compounds, the electrolysis is carried out under current limited control, and high ϕ values can be reached, close to 100%, at the beginning of the electrolysis. These ϕ values are maintained until the electrolysis is under mass transport control, which occurs at about 60 minutes of electrolysis for i values of 5 and 10 mA·cm^−2^. After 60 minutes of electrolysis, ϕ decreases with time. On the other hand, when i is higher than the limiting current density from the beginning of the electrolysis, the electrolysis is mass-transport controlled, ϕ is always less than 100%, and decreases with time, as it is seen in Fig. 12 at 15 mA·cm^−2^.

The maximum ϕ was always reached at the beginning of the electrochemical process (high COD concentrations), then it decreases as the initial COD does. The progressive decay of ϕ can be due to the production of oxidation intermediates difficult to destroy by hydroxyl radicals^70^. Besides, side reactions like oxygen evolution (Eq. 5) and the generation of other oxidants as S_2_O_8_^=^ from the supporting electrolyte would be favored at higher i values which contribute to the decrease of ϕ with the applied current density observed in Fig. 12 ^71^.

## Conclusions

Mineralization of RB5 with Sb-doped SnO_2_ ceramic electrodes in divided and undivided electrochemical reactors has been studied in the present work. In the undivided reactor, high discoloration rates were reached at 595 nm with ceramic electrodes, but new bands appeared indicating the formation of an isoxazole derivative formed by the cyclization of the RB5 molecule in the oxidation process. This fact was not observed for the BDD electrode, which although showed lower efficiency in terms of color removal, led to a 35% of the COD removal at the same electrolysis time.

In the divided reactor, the reduction of the oxidized compounds formed was prevented by the presence of a membrane between the anodic and cathodic compartments. With this reactor configuration, although it was possible the discoloration of the solution while decreasing the COD, the COD degradation was slower than the discoloration rate. Moreover, although the RB5 degradation rate increased with i, the current efficiency decreased with this parameter.
